# Calpains, the proteases of two faces controlling the epithelial homeostasis in mammary gland

**DOI:** 10.3389/fcell.2023.1249317

**Published:** 2023-09-19

**Authors:** Elena R. García-Trevijano, Elena Ortiz-Zapater, Amparo Gimeno, Juan R. Viña, Rosa Zaragozá

**Affiliations:** ^1^ Department of Biochemistry and Molecular Biology, Universitat de Valencia, Valencia, Spain; ^2^ INLIVA Biomedical Research Institute, Valencia, Spain; ^3^ Department of Anatomy and Human Embryology, Universitat de Valencia, Valencia, Spain

**Keywords:** breast cancer, involution, apoptosis, CAPN, adhesion, nucleus, differentiation

## Abstract

Calpain-1 and calpain-2 are calcium-dependent Cys-proteases ubiquitously expressed in mammalian tissues with a processive, rather than degradative activity. They are crucial for physiological mammary gland homeostasis as well as for breast cancer progression. A growing number of evidences indicate that their pleiotropic functions depend on the cell type, tissue and biological context where they are expressed or dysregulated. This review considers these standpoints to cover the paradoxical role of calpain-1 and -2 in the mammary tissue either, under the physiological conditions of the postlactational mammary gland regression or the pathological context of breast cancer. The role of both calpains will be examined and discussed in both conditions, followed by a brief snapshot on the present and future challenges for calpains, the two-gateway proteases towards tissue homeostasis or tumor development.

## Introduction

Calpains are a family of calcium-dependent intracellular Cys-proteases involved in a number of different physiological and pathological processes. Since the first description of calpain in 1964, up-to-date 15 different genes have been described, encoding CAPN1 to 3 and 5 to 16, which are the large catalytic subunit of different isoforms. In addition, two small regulatory subunits with non-catalytic activity, *CAPNS1* and *CAPNS2* have been also identified. The different isoforms have been classified as classical and non-classical calpains, according to the presence or absence of a penta-EF (PEF) hand domain at the C-terminal domain of large subunits and the N-terminal domain of small regulatory subunits. The presence of PEF in classical calpains allows the heterodimerization of both, catalytic and regulatory subunits (for structural review see Sorimachi et al., 2011; Campbell and Davies, 2012; Briz and Baudry, 2017; Nian and Ma, 2021).

Although most of these isoforms (CAPN1, 2, 5, 7, 10 and 13–16) are ubiquitously distributed in mammalian tissues, classical calpain-1 and calpain-2 were the first to be identified and the isoforms best studied in different tissues and pathological conditions. These two isoforms share high sequence homology (Sorimachi et al., 2011) and are known as conventional calpains. Both, calpain-1 and calpain-2 (originally known as µ-calpain and m-calpain, respectively) are formed by heterodimers, consisting of a ∼80 KDa large catalytic subunit (CAPN1 or CAPN2) and a common ∼28 KDa small regulatory subunit CAPNS1 (also, CAPN4), which provides stability to the enzyme (Nian and Ma, 2021).

Conventional calpains are processing rather than degradative enzymes (Nian and Ma, 2021). Indeed, in contrast to other proteases calpains are intracellular proteases that exhibit a limited proteolytic activity on their substrates, acting as regulatory proteases. The end-products of calpains may have functions, protein-protein associations or subcellular distributions different from the corresponding unprocessed substrates (Ono and Sorimachi, 2012; Raimondi et al., 2016; Briz and Baudry, 2017; Miyazaki et al., 2021). However, the governing rules of substrate recognition by calpains are still elusive. Calpains do not recognize a specific sequence in the primary structure of their substrates or a post-translational modification (Sorimachi et al., 2011). Instead, the overall three-dimensional conformation or higher order structures in their substrates have been pointed as the main determinants for substrate recognition (Pariat et al., 2000; Ono and Sorimachi, 2012). Hundreds of substrates have been described as *in vitro* targets of calpain activity, which does not indicate they are necessarily processed by calpains *in vivo*. All in all, calpain-mediated cleavage has been observed in cytoskeleton proteins, membrane-associated proteins, receptors/channels, scaffolding/anchoring proteins, and protein kinases and phosphatases in a variety of tissues and cell types (Croall and Ersfeld, 2007; Ono and Sorimachi, 2012).

Earlier works on the role of calpain system were focused on the activity and *in vitro* regulation of conventional calpains. Since then, several mechanisms have been described to modulate (either activating or inhibiting) their enzymatic activity, such as Ca^2+^ concentration, phosphorylation by ERK1/2 or PKA, binding to phospholipids and acyl-CoA-binding protein, or even to its endogenous inhibitor, calpastatin (CAST) (Ono and Sorimachi, 2012; Nian and Ma, 2021). However, the identity of the calpains end-products and consequently, the isoform-specific functions of both proteases in most tissues is still poorly understood. Are they pro-apoptotic or pro-survival proteases? Do they promote cell proliferation or cell differentiation? Is an isoform limiting the activity of the other or they can compensate each other? Although it is generally accepted that they are not redundant enzymes and that the target-specificity of each isoform depends on their subcellular distribution (Shao et al., 2006; Raynaud et al., 2008; Leloup et al., 2010; Kosenko et al., 2011; Arnandis et al., 2012; Arnandis et al., 2014; Rodríguez-Fernández, 2019; Telechea-Fernández et al., 2018; Rodríguez-Fernández et al., 2021) or the signaling pathways in which they are involved (Wang et al., 2020), a number of reports indicate that the cell type and biological context need to be considered when trying to answer those questions.

Herein we will review the role of conventional calpains in the context of mammary tissue, either under the physiological conditions of the pregnancy/lactation cycle or the pathological breast cancer. Interestingly, these two biological processes share more key regulatory proteins than could be thought at first glance. In fact, much of the molecular signaling regulating the mammary gland homeostasis during the pregnancy/lactation cycle was first identified as oncogenic drivers of breast cancer.

Janus, the classical god of changes and transformations, is represented with two faces symbolizing the uncertainty of what is to come. Likewise, calpain-1 and calpain-2 could be considered the proteases of two faces controlling physiological mammary gland homeostasis, but also promoting breast tumor progression. In this review we will summarize the main findings related to the regulatory and sometimes contradictory role of conventional calpains in these two biological contexts of mammary gland, focused in cell adhesion, cell death and cell proliferation/differentiation. Unfortunately, although an increasing number of reports describe the important functions and effects of calpain activity in different tissues including mammary gland, the specific regulation of calpain distribution and activity to modulate these processes in mammary tissue remains unknown.

## Expression of conventional calpains in the mammary tissue

### The pregnancy/lactation cycle

**FIGURE 1:**
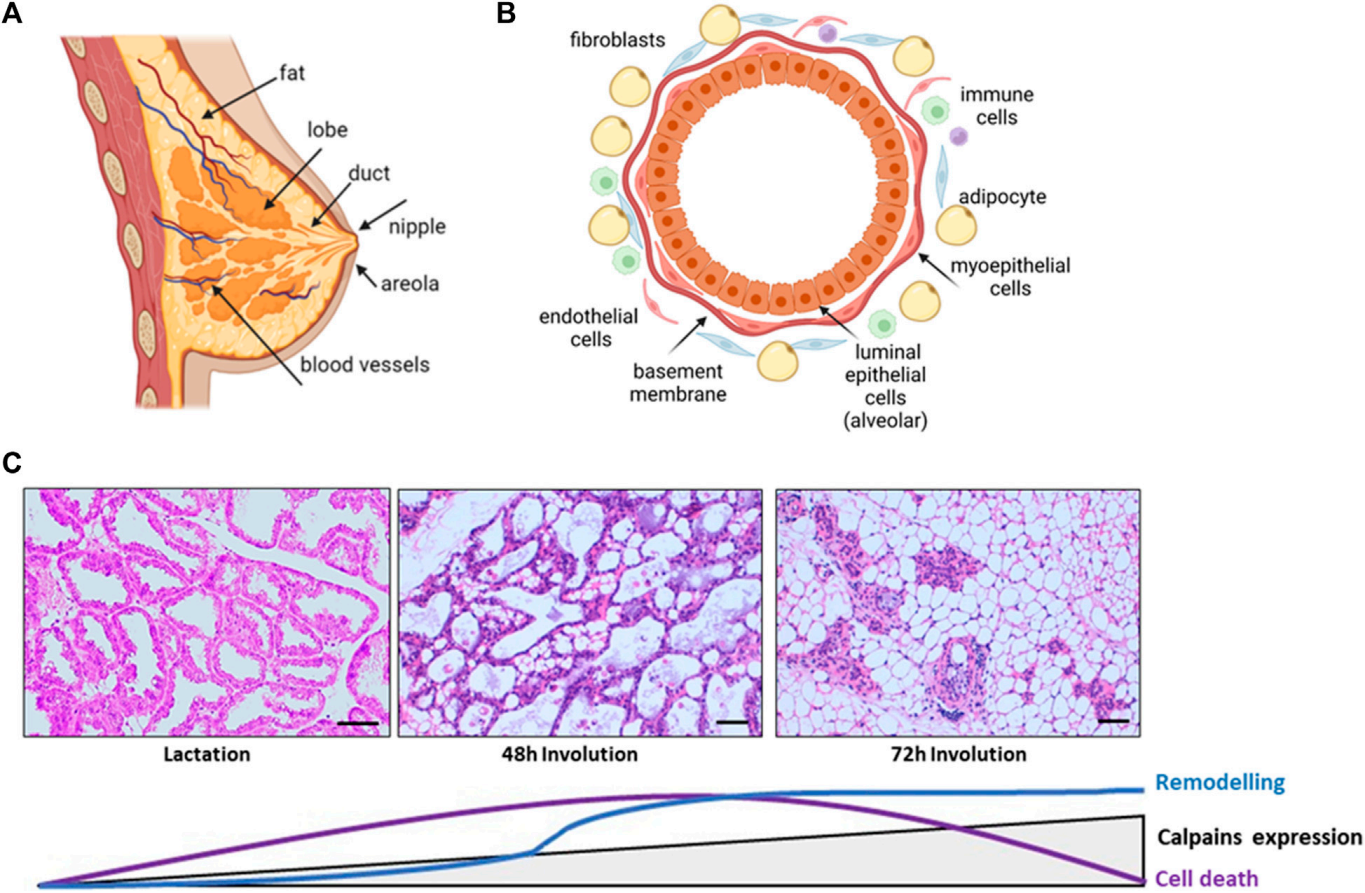
Schematic representation of the structure and morphological changes in the mammary tissue. **(A)** Normal breast anatomy **(B)** Alveolar structure of the gland. Polarized secretory epithelial cells form the lumen of the alveoli and are stretched by contractile myoepithelial cells and basement membrane. Surrounding these glandular structures, the mammary stroma is mainly formed by extracellular matrix, adipocytes, fibroblast, macrophages, and endothelial cells. **(C)** H&E stained sections of lactating and involuting mice mammary gland. The progression of epithelial cell death and remodeling is represented together with the levels of calpains expression. Lactating tissue shows the glandular structure, with several ducts and alveoli tightly closed together, surrounded by extracellular matrix, blood vessels and residual adipocytes. At 48 h weaning, alveoli start to collapse and detached epithelial cells are shed into the lumen. Collapse of alveoli progresses and at 72 h of involution the glandular structures are reduced and adipocytes repopulate the mammary fat pad. Scale bar: 250 μm.

The mammary gland is a complex and specialized tissue whose main function is to synthetize milk, providing nutrition and immunological protection to mammalian offspring (Ward and German, 2004). It is a compound tubule-alveolar gland embedded within an irregular connective tissue known as mammary fat pad (Figure 1A). The glandular epithelial compartment shows two different cell populations, epithelial and myoepithelial, lining ducts, and alveoli. The inner layer of epithelial cells are luminal secretory and ductal cells, undergoing functional differentiation during pregnancy to form the milk-producing secretory acini. The outer myoepithelial/basal cells encasing the luminal cells, are contractile and participate in the delivery of milk in response to oxytocin stimulation. This basal epithelium also harbors stem and progenitor cells, which form both luminal and myoepithelial cells/layer. Finally, the basement membrane separates this epithelial tissue from the surrounding stroma, mainly composed of adipocytes, fibroblasts, macrophages, and other immune and endothelial cells (Stewart et al., 2020; Watson and Khaled, 2020; Biswas et al., 2022) (Figure 1B).

Mammary gland is a unique organ mostly developed after birth, with just the primordia of the gland formed early in embryogenesis. This highly dynamic organ undergoes a series of physiological changes in morphology and function throughout life from menarche to menopause, during each menstrual and pregnancy/lactation cycles (Biswas et al., 2022). At menarche, a rudimental mammary gland is expanded to invade the subjacent mesenchymal tissue creating a more extensive ductal network (Watson and Khaled, 2020). At puberty, the increase in breast size is mainly caused by the accumulation of adipose tissue within the gland. Terminal functional differentiation is reached with the development of alveoli and the synthesis of specific milk proteins late in pregnancy and during lactation. At this point, the mammary gland consists almost entirely of secretory epithelium forming alveolar structures with lumens full of milk fat globules and milk. After weaning, at the end of lactation, there is an extensive regression of mammary tissue in a process known as involution (Wang and Scherer, 2019; Biswas et al., 2022) (Figure 1C).

The process of postlactational involution is finely orchestrated and takes place in two phases in mice. The first stage lasting for 48 h after weaning, is driven mainly by local factors due to milk accumulation within the lumen. This phase is reversible if the suckling stimuli is recovered. Although detectable by regular Western blot, the expression levels of conventional calpains during lactation and the early onset of this phase remain low and constant. Upon weaning, there is a decrease of systemic lactogenic hormones, epithelial cells of the lobulo-alveolar compartment rapidly undergo cell death with increased caspase 3 activation, breakdown of tight junctions, shedding of alveolar dead cells into the lumen and stimulation of a pro-inflammatory environment. A controlled flow of macrophages and other immune cells to the mammary gland would clear dead milk-secreting cells; moreover, some of the survival secretory epithelial cells become phagocytes to remove dying cells from milk (Monks et al., 2005; Atabai et al., 2007). The transcription factor NFκB has been recognized as one of the most important nodes of regulation of mammary gland involution after lactation. This factor known to modulate a number of pro-inflammatory pathways, was shown to be activated in mammary gland and to bind to *CAPN*-1 and -2 gene promoters at 48 h after weaning (Torres et al., 2011). The expression levels of conventional calpains progressively increase from the first phase of involution through the second phase, reaching their higher levels at 72 h involution (Arnandis et al., 2012; Dang et al., 2015). Mammary tissue during this second and irreversible phase is characterized by the proteolytic degradation of the basement membrane and a massive remodeling of the glandular architecture with alveolar collapse, increased number of immune cells within the stroma, and epithelial cell replacement with re-differentiated adipocytes. In the end, mammary gland returns to a pre-pregnant state in preparation for subsequent pregnancies (Landskroner-Eiger et al., 2010; Arnandis et al., 2014; Howard and Lu, 2014; Zaragozá et al., 2015).

From these data and data elsewhere, it can be inferred that all these processes during mammary gland involution must be tightly coordinated by a complex regulatory network where several proteins will play crucial functions as nodes of regulation or as effectors for the final resolution of the biological response (Zaragozá et al., 2015; Biswas et al., 2022) The role of conventional calpains as important effectors for the appropriate physiological resolution of the pregnancy/lactation cycle seems unquestionable. Consequently, dysregulation of calpain-1 and -2 is expected to have important consequences for the homeostasis of this tissue.

### Breast cancer

Altered regulation of conventional calpains have been described in breast tumors or breast cancer cell lines. Indeed, calpain activity affects several signaling pathways related to tumorigenesis (Storr et al., 2011a; Potz et al., 2016; Chen et al., 2019; Nian and Ma, 2021), including cell death/survival pathways, adhesion/migration and invasion, cell cycle control or even the function of different oncoproteins such as HER2 (Pianetti et al., 2001; Kulkarni et al., 2010; Ho et al., 2012; MacLeod et al., 2018). In addition, several studies in HER2^+^ breast cancer cell lines have correlated calpain expression with resistance to chemotherapeutics such as trastuzumab, doxorubicin and cisplatin (Kulkarni et al., 2010; Grieve et al., 2016; Al-Bahlani et al., 2017; MacLeod et al., 2018). Nevertheless, these studies were carried out in cultured cell lines not reflecting the biological conditions found in mammary tissue where calpain expression or activity could be differentially modulated.

Some clinical studies have tried to correlate the clinical outcome of breast cancer patients with calpain protein levels. High calpain-1 protein levels correlated with poor relapse-free survival of HER2+ breast cancer patients treated with trastuzumab following adjuvant chemotherapy (Storr et al., 2011b; Pu et al., 2016). No correlation was found between calpain-2 levels and clinicopathological variables in this group of patients. However, as stated by the authors this *in vivo* study cannot be compared to previous *in vitro* studies since calpain levels in the clinical study were examined before trastuzumab treatment, calpain activity in these patients was not measured and finally, the number of patients included in the study was limited. Therefore, the potential of calpain-1 as a specific biomarker for trastuzumab resistance could not be definitively established.

Additional reports in the literature about the correlation between expression of conventional calpains and relapse-free survival in breast cancer patients result confusing and contradictory at times. Indeed, translational studies in a large cohort of triple-negative and basal-like breast cancer patients found that calpain-1 was not associated with relapse-free survival and identified calpain-2 as the isoform associated with adverse outcomes (Storr et al., 2012). Other clinical study also showed that calpain-1 was overexpressed in breast triple-negative tumors with a significant correlation to lymph node status but not with the other clinicopathological variables, recurrence-free survival, or the overall survival of patients (Al-Bahlani SM et al., 2017). In contrast, high calpain-1 expression was associated with improved disease-free survival of all patients enrolled in a clinical study including different breast cancer subtypes, although improved rates of overall survival were found in the triple negative breast cancer subgroup (Rajković-Molek et al., 2020). In summary, some reports correlate high calpain-1 expression with adverse disease outcome, some with improve disease-free survival and some others found that the levels of calpain-1 did not correlate with any clinicopathological variable. Consequently, a definitive prognostic value for calpains levels in these patients could not be demonstrated (Chen et al., 2019).

It has been argued that discrepancies among reports are most probably caused by the type of analysis and classification of breast tumor subtypes, the limited number of patients in clinical studies and comparison of data obtained *in vitro* and *in vivo* (Storr et al., 2011a; Storr et al. 2011b; Storr et al. 2012; Rajković-Molek et al., 2020; Shapovalov et al., 2022). Calpain activity, protein levels and mRNA levels do not necessarily correlate (Rodriguez-Fernández, 2019; Shapovalov et al., 2022). Importantly, most of clinical reports only study levels of calpain expression, and no data about enzymatic activity or subcellular distribution of calpains in breast cancer patients have been shown (Sorimachi et al., 2011; Storr et al., 2015; Storr et al., 2016). In that sense, early studies showed that calpain-1 is differentially expressed in the peritumoural and intratumoral area of breast cancer patients. Interestingly, only peritumoral calpain-1 expression correlated with relapse-free survival (Storr et al., 2011b). Although the meaning of this observation is unclear, it suggests a complex mechanism of calpains regulation yet to be determined. In addition, calpain activity can be modulated by a number of factors and signaling pathways which could be influenced by the type of tumor and peritumoral area (Ono and Sorimachi, 2012; Nian and Ma, 2021).

Thus, while most authors suggest a pro-tumorigenic role for calpains in cancer progression, discrepancies among reports suggest that calpains may also have anti-tumorigenic roles in different tumor subtypes, phases during cancer progression or context found in the tumoral and peritumoral tissue. In agreement with this, it has been suggested that the effect of overexpression of a specific calpain isoform on breast cancer-survival might depend on the inflammatory context of breast tumors. While high calpain-2 and low CAST expression was associated with improved survival in patients with non-inflammatory breast cancer treated with neoadjuvant chemotherapy, high calpain-1 and high CAST expression in the inflammatory group was associated with improved breast cancer survival (Storr et al., 2016).

The influence of the pro-inflammatory environment on the role of each calpain-isoform is an important aspect to consider in the pregnancy associated breast cancer risk. It has been suggested that the pro-inflammatory environment of mammary gland involution could promote tumor progression (Lim et al., 2010; Torres et al., 2011; Rauner and Kuperwasser, 2021). In addition, the immune tolerance found in mammary gland after weaning has been proposed to also contribute to the neoplastic promotion in mammary tissue (Betts et al., 2018). Many of the regulatory nodes of mammary gland involution, such as STAT3, TGF-β or NFκB, have been identified as persistently activated oncogenes or pro-inflammatory factors favoring neoplasia transformation and metastasis; a notable observation, since as mentioned above, the expression of both calpains is modulated by NFκB. A recent study shows that forced weaning induces morphological changes in the murine mammary gland after short lactation, which were not evident in the long-lactation mice (Basree et al., 2019). Mammary gland from the short-lactation mice exhibited ductal hyperplasia and squamous metaplasia at 4 months after parturition, both preneoplastic conditions for breast cancer and accordingly, a breast cancer risk factor. Moreover, a prevalence study in women showed increased breast cancer risk during the 5 years following parturition (Meier-Abt and Bentires-Alj, 2014). The same study reports that pregnancy-associated increase in breast cancer risk becomes more pronounced with increasing age at first pregnancy.

In the near future, to decipher the potential relationship between calpains dysfunction and pregnancy-associated breast cancer risk, or the isoform-specific role of conventional calpains in breast cancer progression, the subcellular distribution, the cell type and the tissue context need to be considered.

## Role of calpains within the mammary tissue

### Calpains in the modulation of cell adhesion

Conventional calpains have been recognized as key proteases for the regulation of cell adhesion promoting either, epithelial cell clearance during mammary gland involution after lactation or cell migration and invasion during breast tumor progression and metastasis.

Adherens junctions (AJs) are cell-cell adhesion complexes crucial for tissue homeostasis and barrier function of the epithelia. At the cytoplasmic side, AJs are linked to the actin cytoskeleton, stabilizing the epithelium, establishing epithelial cell polarity, and facilitating the cell-to-cell communication needed to regulate cell proliferation and movement. Consequently, disruption of AJs is one of the hallmarks of cancer of epithelial origin including breast carcinoma (Bruner and Derksen, 2018). However, disruption of AJ is not necessarily a pathological condition, but a required mechanism for cell plasticity and tissue reorganization during development, cell proliferation or cell death.

**FIGURE 2:**
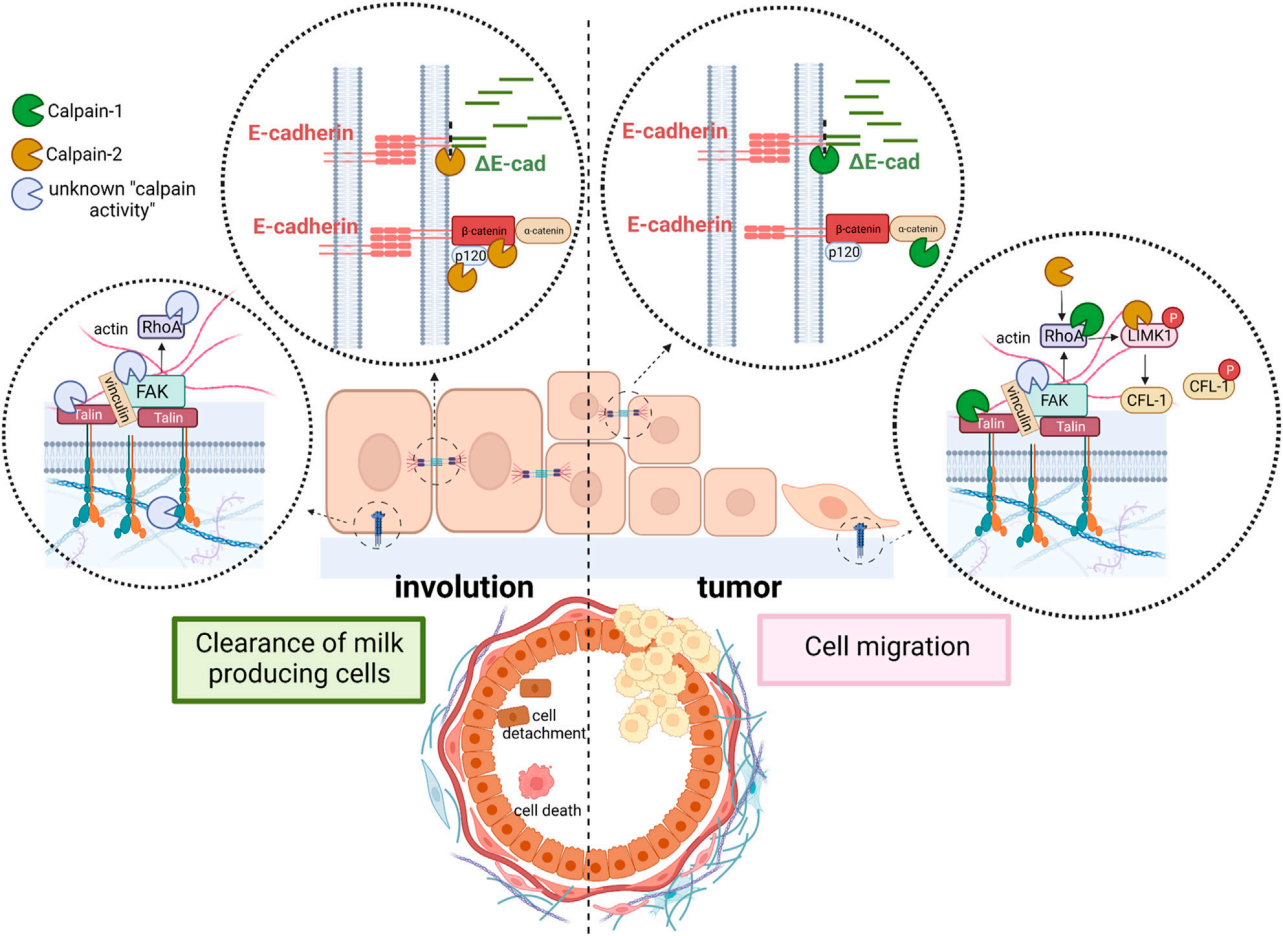
Schematic representation of the context-dependent role of conventional calpains in mammary tissue. Cell adhesion is disrupted during mammary gland involution to clear milk-secreting cells (left). While calpain-2 cleaves E-cadherin and other proteins from AJ, an unknown calpain isoform (most likely calpain-1) cleaves proteins from FA complex. Detached cells are shed into the lumen of alveoli. In contrast, in mammary tumors (right), calpain-1 is the isoform cleaving proteins from both, AJ and FA to induce cell migration and promote invasion and metastasis. In addition, calpain-2-mediated cleavage, and activation of LIMK1 induce phosphorylation and inactivation of the acting severing protein CFL-1, further contributing to cytoskeleton remodeling, cell spreading and mitosis.

One of the major players of cell-cell adhesion during mammary gland development is E-cadherin. E-cadherins are transmembrane receptors with extracellular regions mediating cell-cell adhesion and their intracellular tails interacting with anchor proteins clustered with several actin binding proteins. Both, calpain-1 and 2 are known to target directly or indirectly several proteins from this junctional network, going from E-cadherin, to different anchor proteins and actin binding proteins (Table 1). E-cadherin is broadly expressed in luminal epithelial cells in the mammary gland during all developmental stages, from early embryonic stages to pregnancy or lactation (Bruner and Derksen, 2018). E-cadherin disruption in mammary gland from conditional knockout mice triggers luminal cell apoptosis and cell clearance soon after parturition, preventing the terminal differentiation of milk-producing cells (Boussadia et al., 2002). From a physiological point of view, during mammary gland involution after lactation, disruption of epithelial cell adhesion is an important mechanism to remove undesired secretory cells and to remodel the tissue for the next pregnancy/lactation cycle. Both, calpain-1 and calpain-2 can proteolyze E-cadherin and other adhesion proteins from lactating mammary tissue *in vitro* (Rodríguez-Fernández, 2019). However, according to a cell type and biological context-dependent role of calpains, calpain-2 is the only isoform colocalizing with E-cadherin at epithelial cell membranes during post-lactating mammary gland involution (Figure 2, left). This finding further highlights the context-dependent role of each isoform which will be specifically regulated. Indeed, calpain-2/E-cadherin interaction barely detected at the peak of lactation, increases as the involution progresses. Mice treatment with calpeptin, the inhibitor of calpain activity, was reported to prevent the E-cadherin cleavage during mammary gland involution after lactation (Rodríguez-Fernández, 2019).

**TABLE 1:** Direct targets of calpain activity in mammary gland.

Target	Isoform	Experimental model	References
Cell Adhesion and cytoskeleton pathways			
Cortactin	CAPN1	MDA-MB-231	Hoskin et al. (2015)
	CAPN2	MTLn3	Cortesio et al. (2008)
E-cadherin	CAPN2	Mouse mammary gland	Rodríguez-Fernández (2019)
	CAPN1	MCF-7, BT-474	Rodríguez-Fernández (2019)
	n.s.	MCF-7	Rios-Doria et al. (2003)
Ezrin	CAPN2	TS/A	Lee and Shyur (2012)
FAK	CAPN1	MDA-MB-231	Hoskin et al. (2015)
	CAPN2	MCF-7	Li et al. (2017)
	n.s	BT20, MDA-MB-231	Xu et al. (2010)
	CAPN2	TS/A	Lee and Shyur (2012)
	CAPN2	MCF-7, T47D	Libertini et al. (2005)
	CAPN1	MCF-7	Hou et al. (2012)
Fodrin	n.s.	MCF-7	Sareen et al. (2007)
	CAPN1	MDA-MB-231	Al-Bahlani et al. (2017)
α-Spectrin	n.s.	MCF-7	Rios-Doria et al., 2003, 2004
LIMK1	CAPN2	MDA-MB-231	Rodríguez-Fernández et al. (2021)
Paxillin	n.s	BT20, MDA-MB-231	Xu et al. (2010)
Pp60c-Src	n.s.	MDA-MB-231	Xu et al. (2010)
	n.s.	MDA-MB-435	Tan et al. (2005)
PTPμ (PTP1B)	CAPN2	MTLn3 cells	Cortesio et al. (2008)
Talin	n.s.	Mouse mammary gland	Rodríguez-Fernández (2019)
	CAPN1	MDA-MB-231, MDA-MB-468, MCF-7, BT-474	Rodríguez-Fernández (2019)
	CAPN1	MDA-MB-231	Hoskin et al. (2015)
	n.s.	MDA-MB-231	Xu et al. (2010)
Vimentin	n.s.	MDA-MB-231, Hs578T	Kim et al. (2017)
β-catenin	n.s.	MCF-7	Rios-Doria et al. (2004)
	n.s.	Mouse mammary gland	Rodríguez-Fernández (2019)
	CAPN1	MCF-7, BT-474	Rodríguez-Fernández (2019)
δ-catenin (p120)	n.s.	Mouse mammary gland	Rodríguez-Fernández (2019)
	CAPN1	MCF-7, BT-474	Rodríguez-Fernández (2019)
Epithelial cell death pathways			
Bax	n.s.	MCF-7	Sobhan et al. (2013)
	CAPN1	MCF-7	Gao and Dou (2000)
Bid	n.s.	MDA-MB-231, MCF-7	Mandic et al. (2002)
Bcl-2	n.s.	MCF-7	Pozo-Guisado et al. (2005)
Caspase-12	CAPN1	MDA-MB-231	Al-Bahlani et al. (2017)
Lamp2a	CAPN1	Mouse mammary gland	Arnandis et al. (2012)
VATB2	n.s.	Mouse mammary gland	Arnandis et al. (2012)
Nup62	CAPN1	Mouse mammary gland	Arnandis et al. (2014)
PARP	CAPN1	MCF-7	Tagliarino et al. (2003)
	n.s.	MCF-7 and T47D	Pink et al. (2000)
	n.s.	MCF-7	Cui et al. (2007)
p53	CAPN2	MCF-7, T47D	Libertini et al. (2005)
PP2A (B56)	n.s	MCF-7	Bertoli et al. (2009)
RelA	n.s.	MCF-7	Fei et al. (2013)
PMCA1a/b	n.s.	MCF-7	Sareen et al. (2007)
Proliferation/Differentiation pathways			
Cyclin E	CAPN2	MCF-7, T47D and xenografts	Libertini et al. (2005)
	CAPN1	MCF-7	Hou et al. (2012)
	n.s.	ZR-75	Wang et al. (2003)
Cyclin D1	n.s.	MDA-MB-231	Choi et al. (1997)
p21^waf1/cip1^	n.s.	MCF-7	Khan et al. (2002)
Histone H3	CAPN1	Mouse mammary gland	Arnandis et al. (2014)

n.s. CAPN isoform was not specified. Name of breast cell lines is indicated.

Calpain-mediated cleavage of E-cadherin has been shown to disrupt its interaction with its anchor proteins β-catenin and p120, promoting the disassembly of protein complexes crucial for cytoskeleton function (Rios-Doria et al., 2003; Bruner and Derksen, 2018). In addition, proteolysis of anchor proteins is also known to decrease the stability of E-cadherin at AJs (Davis et al., 2003). Interestingly, both β-catenin and p-120 are also targets of calpain activity during mammary gland involution (Rodríguez-Fernández, 2019), an event which further assures the complete disassembly of AJs during mammary gland remodeling (Figure 2, left). However, a fast AJ reassembly should be guaranteed for the next pregnancy/lactation cycle.

Cleavage of E-cadherin induced by a number of stress stimuli, including high calcium concentration and its accumulation in cultured medium was long ago reported in mammary tumor cells (Wheelock et al., 1987). E-cadherin undergoes endocytosis when AJs are disrupted. It seems that the fate of truncated E-cadherin (ΔE-cadherin) is not necessarily degradation, but will rather depend on whether it is bound to AJs or is a free E-cadherin-complex (Bruner and Derksen, 2018). Calpain-2/ΔE-cadherin complex, analyzed by PLA assay in mammary gland during postlactating involution, was not degraded but instead accumulated in the cytoplasm of epithelial cells (Rodríguez-Fernández, 2019). The cytoplasmic accumulation of several forms of ΔE-cadherin as a result of calpain proteolytic activity was also demonstrated in breast and prostate carcinoma cell lines as well as in several types of adenocarcinoma (Rios-Doria et al., 2003). Although a soluble ΔE-cadherin product of calpain activity has been proposed to have prognosis value for breast cancer (Hofmann et al., 2013), the role of the cytosolic ΔE-cadherin accumulation in breast epithelial cells has not been completely elucidated.

Although mammary gland-specific depletion of E-cadherin did not develop tumors in knockout mice (Boussadia et al., 2002), several evidences indicate that inhibition of E-cadherin function is sufficient to induce invasion of cancer cells (Bruner and Derksen, 2018). It has been hypothesized that tumors will not develop unless the pro-tumorigenic event that induces the loss of E-cadherin is preceded or occurs concomitantly with the loss of protecting signals such as p53 or PTEN (Bruner and Derksen, 2018). Consistently, the combined loss or inactivation of E-cadherin/p53 or E-cadherin/PTEN in mammary gland leads to the development of invasive lobular carcinoma (Libertini et al., 2005; Annunziato et al., 2016). However, it seems that alternative mechanisms to disrupt E-cadherin function must contribute to the invasive phenotype; a fraction of patients with invasive lobular carcinoma retain E-cadherin expression and 50% of patients showing loss of E-cadherin do not have *CDH1* inactivating mutations (Bruner and Derksen, 2018). According to a putative role of calpains in these alternative mechanisms, calpain-1 has been shown to disrupt cell-cell adhesion and promote cell migration in breast cancer cells (Rios-Doria et al., 2003; Rodríguez-Fernández, 2019) (Figure 2, right). In addition, both p53 and PTEN are well known targets of calpain activity in breast cancer cells as well as in other cell types (Kubbutat and Vousden, 1997; Libertini et al., 2005; Kulkarni et al., 2010). Interestingly, although calpain-1 has been described to proteolyze E-cadherin in breast cancer cell lines (Annunziato et al., 2016; Rodríguez-Fernández, 2019), it is not involved in the physiological cleavage of E-cadherin during mammary gland involution after weaning. The pro-tumoral environment might trigger the molecular switch from the physiological calpain-2/E-cadherin to the metastatic calpain-1/E-cadherin-cleavage observed in breast cancer cell lines. It is tempting to speculate that the fate of ΔE-cadherin might be conditioned by the calpain-isoform inducing its cleavage. After cleavage by calpain-2 in physiological conditions, ΔE-cadherin is internalized and stored in the cytoplasm. This mechanism would allow the remaining epithelial cells in the tissue to re-express cadherin at the cell surface and to re-establish AJs in a more efficient manner, a mechanism predicted to have important implications during mammary gland remodeling after lactation.

In addition to AJs, the presence of integrin receptors and the cytoplasmic proteins that form the focal adhesion (FA) complexes also contribute to the process of cell detachment or migration. Calpains are long ago known to be involved in FA turnover. An increasing number of reports identify different calpain targets either in integrin clusters, FAs, or downstream pathways (Bialkowska et al., 2000; Franco and Huttenlocher, 2005; Paavolainen and Peuhu, 2021) (Table 1).

Integrin β3, described to be the target of calpain activity is an essential integrin for lobulo-alveolar differentiation of mouse mammary gland. Moreover, integrin clusters seem to be dependent on calpain activity for their formation (Bialkowska et al., 2000). Talin is a key protein from FAs that directly connects integrins to the actin cytoskeleton and is the protein from FA complexes most frequently reported to be a calpain target in different tissues (Franco and Huttenlocher, 2005; Storr et al., 2011a; Chen et al., 2019; Nian and Ma, 2021). Mutation of talin at its calpain cleavage site skipped proteolysis in response to increased Ca^2+^ influx, but most importantly, it also attenuated the degradation of the other proteins from the FA complex which retained their interaction (Chang et al., 2017). During mammary gland involution talin-1 was shown to be the target of calpain activity (Figure 2, left), although the precise calpain isoform involved in such cleavage has not been identified yet (Rodríguez-Fernández, 2019). However, in either luminal or triple negative breast carcinoma cell lines talin-1 was proteolyzed only by calpain-1 (Rodríguez-Fernández, 2019) (Figure 2, right). Since calpain-2 protein levels are much higher than calpain-1 levels in triple negative breast cancer cell lines, the latter observation suggests that the isoform-specific role of calpains in FAs is not dependent on calpain levels but on the cell context regulation of its activity or subcellular distribution. In agreement with this, in other cell types such as fibroblast, calpain-2, but not calpain-1, is required for proteolysis of talin (Franco et al., 2004; Franco and Huttenlocher, 2005).

RhoA is another downstream effector of FA described to be the target of both calpains and crucial for the modulation of cell spreading and morphology (Figure 2, right). Although not specifically studied in mammary gland, the resultant effect of RhoA cleavage is again dependent on the cell type and context. Calpain-1 cleavage of the RhoA C-terminal domain inhibits integrin-induced actin filament assembly and cell spreading in endothelial cells (Kulkarni et al., 2002). The same effect was observed in cultured fibroblast where calpain-1 degrades a stable and functional N-terminal-RhoA fragment produced by serin proteases (Girouard et al., 2016). On the contrary, calpain-2 promotes mTOR/ROCK-RhoA pathway and actin polymerization through the cleavage and inactivation of PTEN in rat hippocampus (Briz and Baudry, 2017). Nevertheless, since RhoA activity is tightly regulated through several mechanisms (De Seze et al., 2023), is not surprising that calpain activity on different targets might either induce or block RhoA activity in specific cell types and conditions. In this sense, Piezo channels, functionally expressed in malignant breast cancer cell lines, mediate Ca^2+^-influx to activate RhoA by a calpain-dependent mechanism regulating the formation and orientation of FAs (Pardo-Pastor et al., 2018). RhoA is known to induce the phosphorylation of the actin-severing protein cofilin-1 (CFL-1) through the activation of ROCK/RhoA/LIMK1 pathway (Briz and Baudry, 2017). However, recent data suggest a calpain-2-mediated cleavage of LIMK1 as a novel RhoA-independent mechanism for LIMK1 activation and CFL-1 phosphorylation in breast cancer cell lines (Rodríguez-Fernández et al., 2021) (Figure 2, right).

All in all, these data highlight the important role played by conventional calpains on cell adhesion disruption and actin dynamics. Depending on the cell type and context, cleavage of adhesion complexes or their downstream effectors by specific calpain isoforms will lead to a different outcome; while cell adhesion disruption will result in cell death during mammary gland involution, in breast cancer cells it will promote cell migration. Moreover, the role of calpains on cytoskeleton organization and cell adhesion might be extended to other biological and pathological processes modulated by actin-dynamics yet to be determined.

### Calpains functions in epithelial cell death

Following milk stasis, mammary involution is the process by which senescent mammary cells are cleared, the lobuloalveolar structures regress and the gland returns to a pre-pregnant state. Among the signaling pathways regulating this programmed cell death both, STAT3 and NFκB are essential (Watson, 2006; Torres et al., 2011; Zaragozá et al., 2015; Watson and Khaled, 2020). As mentioned before, conventional calpains are NFκB target-genes upregulated early during the weaning process. Calpains will then propagate the response proteolyzing various substrates to promote cell apoptosis (Table 1); indeed, several caspases such as caspase-7, -9, -10 and -12 have been identified as calpain-targets (Storr et al., 2011a; Nian and Ma, 2021). Besides, activated calpains are involved in endoplasmic reticulum-mediated apoptosis (Storr et al., 2016), and in the mitochondrial apoptotic pathway through the cleavage of proteins from the Bcl-2 family (Storr et al., 2011a; Nian and Ma, 2021). Both conventional calpains are known to cleave the N-terminal domain of Bcl-2, Bax, and Bid proteins and these truncated forms translocate to the mitochondria where they induce mitochondrial permeabilization and the release of cytochrome c and apoptosis inducing factor (AIF) to the cytosol. This mitochondrial leakiness will lead to the activation of caspase-3 and initiate apoptotic execution. In fact, mitochondrial fractions incubated with either recombinant Bcl-2 or Bid showed only very low cytochrome c release whereas incubation of mitochondria with calpain-truncated Bcl-2 or Bid induced substantial or almost complete release of cytochrome c (Gil-Parrado et al., 2002). Similarly, Bax cleavage generates a potent proapoptotic 18 kDa fragment that does not interact with the antiapoptotic Bcl-2 protein and mediates cytochrome c release (Gao and Dou, 2000). It is noteworthy that calpain proteolysis of Bcl-2 transforms it from an anti- to a pro-apoptotic molecule whereas the proapoptotic proteins Bax and Bid become even more active in their calpain-truncated forms.

**FIGURE 3:**
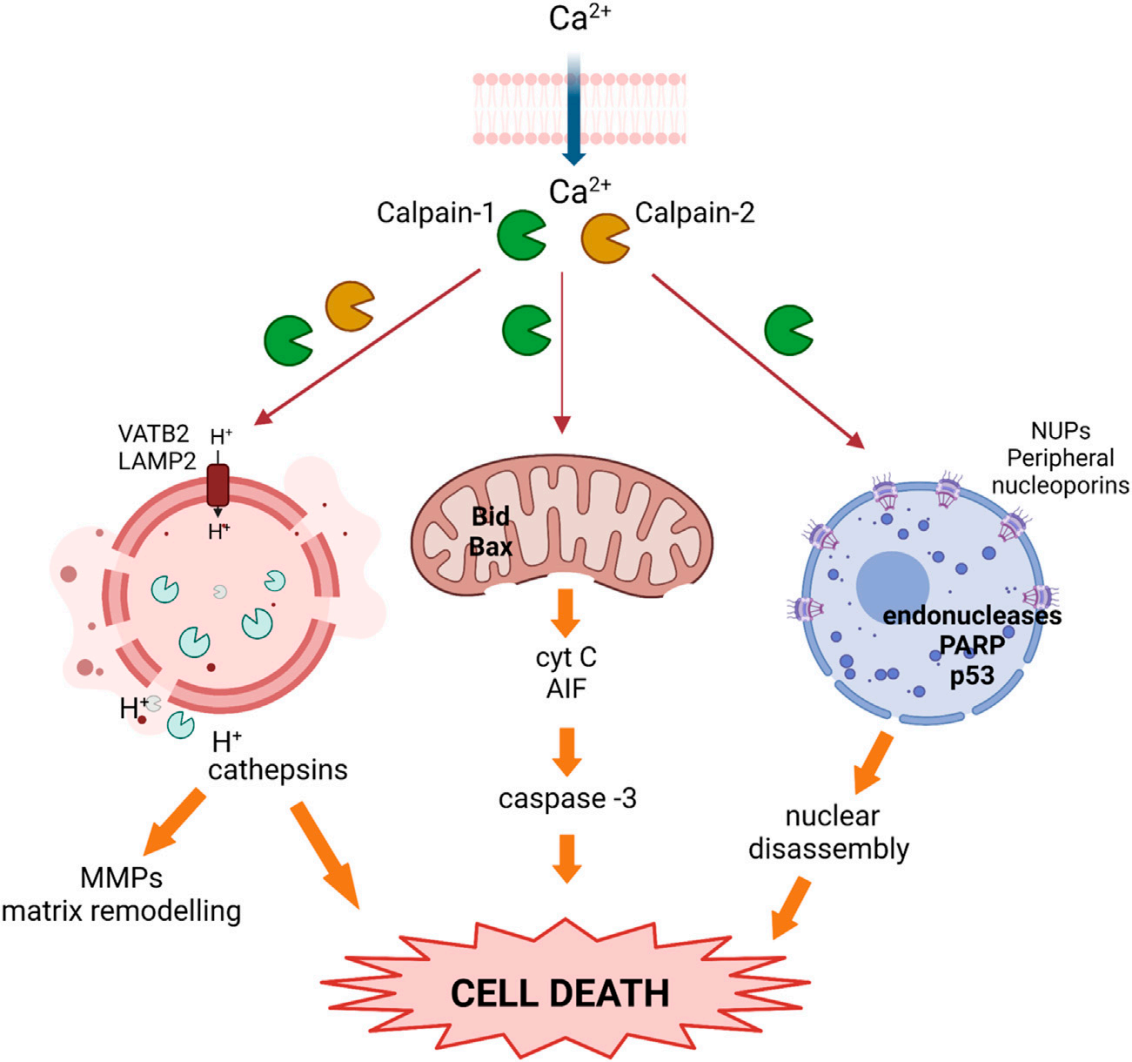
Activated calpains trigger cell death during the weaning process. Upon calcium overload, calpains become activated and translocate to different subcellular organelles where they cleave target proteins, inducing nuclear, lysosomal, and mitochondrial membrane destabilization, the release of cathepsins and pro-apoptotic proteins, and prompting cell death.

In the murine mammary gland, initial studies on the mechanism of cell death during involution focused on the activation of the intrinsic apoptotic pathway, characterized by mitochondrial outer membrane permeabilization, release of cytochrome c and other proapoptotic factors (Zaragozá et al., 2005; Watson, 2006; Kreuzaler et al., 2011; Wang and Scherer, 2019). It has been demonstrated that calpain-1 is involved in this mitochondrial proapoptotic pathway in either, physiological or pathological mammary gland (Arnandis et al., 2012; Sobhan et al., 2013; Al-Bahlani et al., 2017; Ciscato et al., 2020). Indeed, calpain-1 is present in mitochondrial fractions in both, lactating and involuting mammary gland; however, its protease activity increases as weaning progresses, presumably due to the cytosolic increase in calcium levels as a result of milk stasis, reaching its highest level at 72 h involution. Concomitant with calpain-1 activation at the mitochondria, there is cytochrome c release from mitochondrial fractions to the cytosolic compartment during involution, suggesting that calpain-1 is the major player in mitochondrial destabilization (Figure 3) (Arnandis et al., 2012). Similarly, in MCF-7 treated with the proapoptotic drug zerumbone, calpain activity is required for Bax activation preceding the mitochondrial permeabilization and caspase-dependent cell death (Sobhan et al., 2013). Other studies have shown that cisplatin-induced apoptosis of triple negative MB-231 breast cancer cells takes place through the calpain-1-mediated cleavage of caspase-12. Cisplatin treatment induced endoplasmic reticulum stress and structural changes in mitochondria in a concentration-dependent manner. In contrast, calpain-1 silencing or calpeptin treatment, attenuated cisplatin-induced apoptosis in these cells (Al-Bahlani et al., 2017). However, other studies in human and murine breast cancer cells, have suggested that mitochondrial permeabilization and mitochondrial calcium overload are a priming event needed for calpain activation and induction of cell death (Sareen et al., 2007; Ciscato et al., 2020). Nonetheless, the role of calpain-1 on the mitochondrial death pathway described in mammary gland seems to be common to other tissues. Calpain-1 found in mitochondrial-enriched fractions from ischemic neurons (Cao et al., 2007) cleaves substrates such as the Na^+^/Ca^+2^ exchanger NCX3 in the inner mitochondrial membrane, causing mitochondrial calcium overload and release of cytochrome c and apoptosis-inducing factor (Kar et al., 2009; Norberg et al., 2010; Chelko et al., 2021). Whatever the sequence of events that occur in the mitochondrial membrane, these studies pinpoint the importance of the subcellular location of calpains for their subsequent activation, substrate accessibility and final fate of the cell.

Apart from this mitochondrial apoptotic pathway, several findings in the involution process pointed out that there were other mechanisms in the mammary tissue early in involution that also led to programmed cell death of epithelial cells. Alteration of nuclear morphology is a common feature shared by different cell death programs. In this sense, calpains translocate to the nuclear membrane during mammary gland involution affecting nuclear pore complexes and, thus, nuclear membrane permeability (Arnandis et al., 2014) (Figure 3). Conventional calpains were present in nuclear fractions after 72 h involution; immunofluorescence or immunoprecipitation analysis showed that both proteases interacted with several nucleoporins that form the nuclear pore complex. Indeed, it was demonstrated that calpains cleave several peripheral nucleoporins during involution, affecting the structure of nuclear pore complexes with the subsequent impairment of nuclear transport selectivity. Calpain activity and location within the nucleus has already been described in other tissues, causing altered permeability of the nuclear membrane and cell death (Bano et al., 2010; Chang et al., 2015; Sheng et al., 2015). Alteration of the nuclear envelope may have a key role in the redistribution of death-inducing factors, in a positive amplification loop that would contribute to cell death and disassembly. Supporting the cell-context dependent role of calpains, their nuclear targets in transformed breast cancer cells are not at the nuclear pore complex but in the nucleoplasm. Induced apoptosis in MCF-7 or MD-468 breast cancer cells was shown to be mediated by calpain-1 translocation into the nucleus. Upon calpain-dependent endonuclease activation, PARP and p53 were proteolytically cleaved, leading to DNA fragmentation and apoptosis (Tagliarino et al., 2003; Cui et al., 2007). However, given the pleiotropic role of calpains, these proteases may have contradictory roles in the cell nucleus. Studies in non-transformed mammary MCF10A found that the calpain-2-mediated cleavage of nuclear Ku80 could be a mechanism of resistance to induced-DNA double-strand breaks (Baek et al., 2016). In contrast, calpain-2 played an important role in the nucleocytoplasmic trafficking of forkhead box protein P1 (FOXP1) via the PI3K-AKT pathway in breast cancer patients; cytoplasmic relocalization of FOXP1 correlated with reduced overall survival in breast invasive ductal carcinoma patients (Yu et al., 2018).

On the other hand, the relevance of calpain in the modulation of other cell death pathways during mammary gland involution have been also studied. Mitochondrial or nuclear permeabilization are not the only pathways to be modulated by calpain activity to induce cell death. Further studies on murine mammary gland involution showed that during the involution first phase, luminal alveolar cells also die via a lysosomal-mediated pathway (Kreuzaler et al., 2011; Arnandis et al., 2012). Lysosomal activity is essential to preserve cellular homeostasis in mammary gland, and lysosomal membrane permeabilization results in massive release of the lysosomal contents into the cytosol (Kreuzaler et al., 2011; Lloyd-Lewis et al., 2018). Therefore, the lysosomal-mediated cell death is triggered by disruption of lysosomal membrane stability. The role of calpains in the lysosomal-mediated death pathways during mammary gland involution has been studied. It has been demonstrated that calpain activity in lysosomal-enriched fractions increased by twofold after 24 h weaning and remained elevated thereafter, leading to lysosomal destabilization and the release of lysosomal proteases into the cytosolic compartment (Figure 3) (Arnandis et al., 2012). Lysosomal-membrane integrity is ensured by several membrane proteins such as HSP70, the glycoproteins LAMP1 and LAMP2 (Eskelinen, 2006) or the vacuolar-type H^+^-ATPase (V-ATPase). Cleavage of these proteins will destabilize the lysosomal membrane and induce cell death. The identification of calpain targets in lysosomal fractions of involuting mammary gland revealed the mechanisms of the calpain-mediated destabilization of lysosomes. Indeed, it was observed that as involution progressed, calpain-1 and calpain-2 translocated from the cytosol to the lysosomal membrane where they degraded the cytosolic tail of LAMP2A and the subunit b of the vacuolar-type proton ATPase. Furthermore, calpain-1 silencing with siRNA prevented LAMP2A degradation in 72 h weaned mice (Arnandis et al., 2012).

The consequences of the calpain-mediated destabilization of lysosomes have been reported. Activation of STAT3 in mammary tissue during involution upregulates the expression of cathepsins B and L which are known lysosomal proteases (Kreuzaler et al., 2011). Calpain-mediated lysosomal destabilization triggers the release of cathepsins (Zaragozá et al., 2009; Margaryan et al., 2010). Since these cathepsins will act on downstream targets such as MMP-9, calpains will expand the signaling cascade that leads to epithelial cell death and mammary tissue remodeling through lysosomes-leakiness.

Lysosomal weakness that involves cathepsins release is a known pathway to be targeted in breast cancer cells (Ostenfeld et al., 2005) and, based on current knowledge, one could hypothesize that calpains are key mediators in this lysosomal cell death. VATB2, identified as a calpain-target, is crucial for lysosomal-mediated cell death. In breast and gastric cancer cell lines, inhibition of the V-ATPase causes lysosomal dysfunction and induces apoptosis (McHenry et al., 2009; Chen et al., 2022), sensitizing cancer cells to chemotherapy (Piao and Amaravadi, 2016; Dong et al., 2022). Nevertheless, the release of lysosomal content, such as cathepsins B and D, initiates a cascade of cell signaling events that may not always lead to cell death. Under specific circumstances cell fate can be the opposite and lysosomal leakage may be associated to cell survival, as it is the case for cancer cells, in which partial release of lysosomal cathepsins has a key role in tumor progression. Indeed, V-ATPases participate in the invasion and metastasis of tumor cells facilitating cathepsins activation and release; a process associated with cell invasion through matrix metalloproteinase activation (Jung et al., 2021).

All these studies in mammary gland involution and breast cancer emphasize the complexity of the calpain system. As inferred from the information previously given, calpains can be both, proapoptotic or survival factors depending on cellular context, type of apoptotic stimuli and subcellular localization of the protease (Tan et al., 2006). Although several studies in breast cancer cells have shown that calpain activation and mitochondrial dysfunction are key mechanisms for the cytotoxicity of different pharmacological anticancer drugs (Tagliarino et al., 2003; Cui et al., 2007; Sareen et al., 2007; Sobhan et al., 2013; Al-Bahlani et al., 2017; Ciscato et al., 2020), calpain activity has also been implicated in the pro-survival activity of NFкB or p53 in cancer cells (Pianetti et al., 2001; Pozo-Guisado et al., 2005; Fei et al., 2013). Interestingly, these two calpain-targets are crucial regulatory nodes for mammary gland involution, (Zaragozá et al., 2009; Torres et al., 2011); indeed, this process is delayed in the absence of a functional p53 gene (Jerry et al., 1999; Jerry et al., 2002) or NFκB (Connelly et al., 2010). Once again, it has been remarked the multi-faceted role of calpains in diverse signaling pathways. The findings presented herein highlight the context-dependent and opposing pro-survival or pro-apoptotic roles of conventional calpains, though further research is needed to elucidate the precise mechanisms and the specific isoforms playing a particular role in each cellular context.

### Role of calpains in proliferation/differentiation in mammary gland

Cell proliferation and differentiation take place in different cell types throughout the whole pregnancy/lactation/involution cycle. Some of the signaling pathways involved in the latter processes are also triggered and altered during breast tumorigenesis. Calpains are long known to be involved in the process of cell differentiation and proliferation. The important function of calpains in those processes was reported in early experiments where calpain inhibitors such as calpeptin and other thiol protease inhibitors were shown to restrict cell cycle progression or reduce the growth rate of transformed and non-transformed mammalian cells in response to a number of stimuli. Exogenous overexpression of CAST, or depletion of specific calpain isoforms facilitated the identification of calpain substrates as well as those signaling pathways modulated by calpains during cell proliferation. Calpain activity promotes the cell cycle progression through the modulation of key proteins for G1 restriction checkpoint, such as Cyclins E, D1, p21 (waf1/cip1), CDKs or RB. In addition, calpains also have an important function in other phases of cell cycle (Table 1) (Nian and Ma, 2021).

**FIGURE 4:**
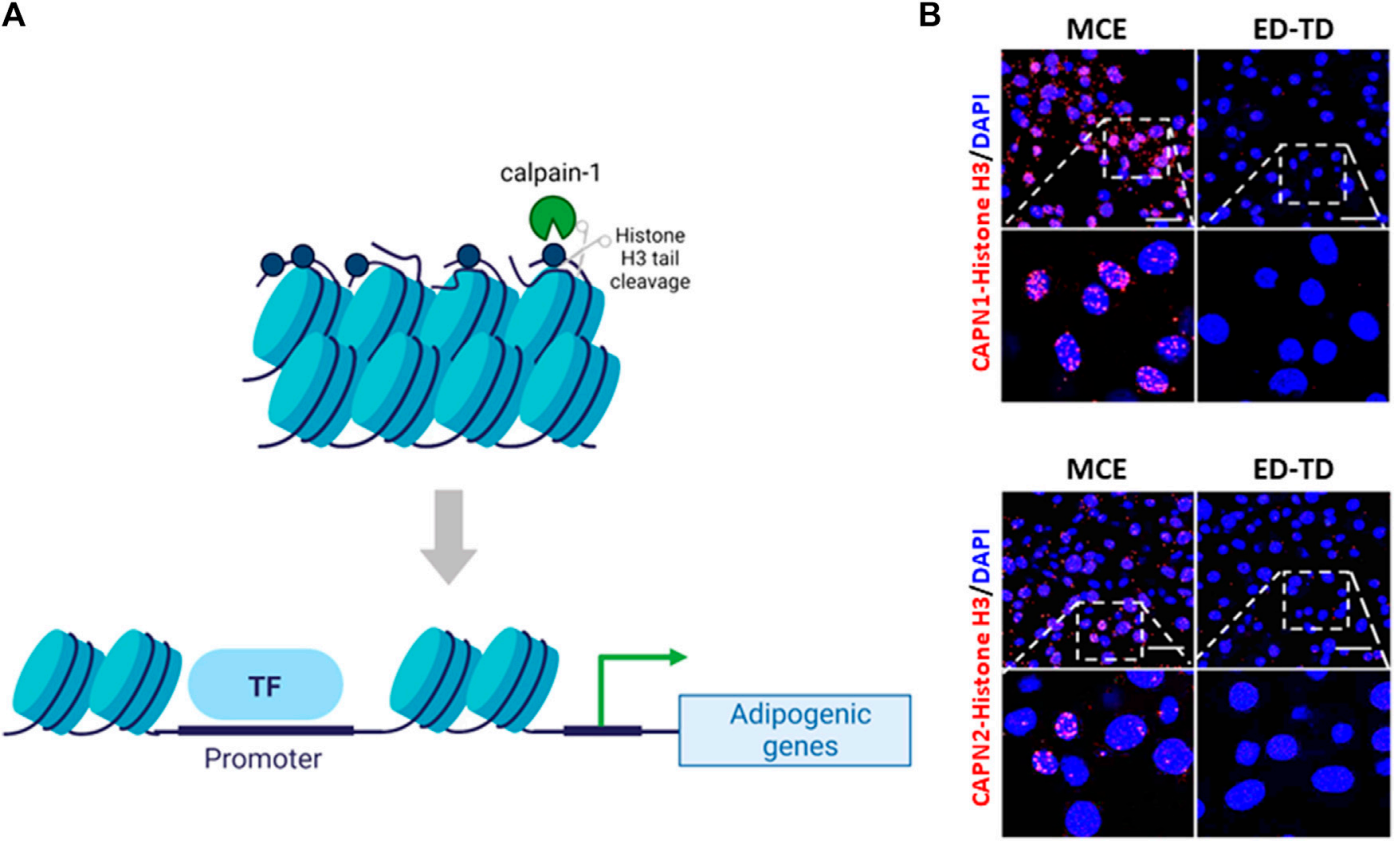
Calpains and chromatin interaction during pre-adipocyte differentiation. **(A)** Schematic representation of the epigenetic cleavage of Nt-histone H3 by calpain-1 in adipocytes. During mammary gland involution calpain-1 binds to and cleaves Nt-histone H3 on adipogenic gene promoters inducing chromatin relaxation and gene expression **(B)** Representative images of CAPN1 and CAPN2 interaction with Nt-histone H3 during the differentiation of 3T3-L1 cells are shown. CAPNs/Nt-histone H3 interaction was analyzed by PLA (red) during pre-adipocyte monoclonal expansion (MCE) and transition from early to terminal differentiation (ED-TD). Nuclei were counterstained with DAPI (blue). Scale bar, 21 μm. (Nt: N-terminal tail).

Unfortunately, the role of calpains in epithelial cell proliferation and differentiation during pregnancy or lactation has not been studied yet. However, 3D studies in MCF-10A non-tumoral breast cell line to mimic structures that resemble the acini of human breast, revealed that the architecture of acini derived from CAPNS1 depleted cells is altered (Raimondi et al., 2016). Although not identifying the specific isoform, the authors conclude that calpains may play an important role in the initiation of the differentiation process in this system. Nevertheless, during mammary gland involution after lactation most cells undergoing proliferation/differentiation are not epithelial but stromal cells. In fact, most of the non-lactating mammary gland mass consists of stromal adipose tissue (Landskroner-Eiger et al., 2010). During the second phase of involution, after epithelial cell death, the basement membrane and ECM break down and, dedifferentiated adipocytes proliferate and re-differentiate back into mature adipocytes to repopulate the mammary fat pad (Wang and Scherer, 2019). Calpain-1 was shown to be localized in the nucleus of dedifferentiated adipocytes during the second phase of involution (Arnandis et al., 2014). Conversely to its subcellular distribution in epithelial cells, nuclear calpain-1 was not found to be associated to the nuclear pore complex, but interacting with histone H3. This calpain-1/histone H3 interaction seems to be part of the differentiation program of pre-adipocytes repopulating the mammary fat pad during involution. Analysis of adipocyte fractions from involuting mammary gland as well as *in vitro* enzymatic assays showed that calpain-1 was the specific isoform cleaving the N-terminal tail of histone H3. Although the functional consequences of calpain-1-mediated cleavage of histone H3 are unknown, it is tempting to speculate that this cleavage might be an epigenetic signature for selected genes upon adipocyte differentiation. In this sense, differentiating preadipocytes have been reported to experience important epigenetic changes in the nuclear compartment affecting chromosome positioning (Kuroda et al., 2004), promoter interactions prior to adipogenic genes activation and expression (He et al., 2018) and chromatin remodeling (Salma et al., 2004). In agreement with this, a full colocalization of calpain-1 and the euchromatin/active marker H3K4me3 was observed in tissue sections from mammary gland involution (Arnandis et al., 2014). Interestingly, this colocalization was exclusively observed in stromal cells. In addition, it is well-established that adipogenic gene promoters are marked by early changes in histone modification patterns (Macchia et al., 2021) to keep an opened chromatin structure accessible to transcription factors. Accordingly, cleavage of the histone H3 tail might result in a decondensed chromatin structure in those adipogenic genes which need to be expressed during the differentiation process (Figure 4A). Calpain-1 was found to bind to the C/EBPα and leptin gene promoters, two adipogenic genes involved in terminal differentiation and the acquisition of the adipocyte phenotype, respectively. Consequently, the expression of these two genes was increased in mammary stroma during involution compared to the lactating mammary gland (Arnandis et al., 2014).

Interestingly, further reinforcing the idea of a context-dependent role of conventional calpains, observations from our group (Rodríguez-Fernández, 2019) indicate that both calpain isoforms interact with N-terminal tail of histone H3 during the differentiation of 3T3-L1 pre-adipocytes (Figure 4B). However, their distribution during preadipocyte mitotic clonal expansion is completely different. Calpain-1 was observed surrounding condensed metaphasic chromosomes, but not colocalizing with them, and at telophase, it was widely distributed into the cytosol. In contrast, calpain-2 was found either colocalizing with the prometaphasic chromosome rosette, surrounding chromosomes along the metaphase plate or concentrated in the whole nucleus during telophase. These data suggest that although not observed in mammary gland, both calpains might have different and important functions for adipocyte differentiation. In that sense, it has been proposed that calpain-2 limits the activity of calpain-1 (Shinkai-Ouchi et al., 2020) and consequently, the pattern of activation of each isoform might be sequential. However, this hypothesis has not been demonstrated *in vivo* and in addition, the inflammatory component from mammary gland involution, which is absent in cultured cells, might condition the behavior of both calpains. Although this epigenetic mark was exclusively observed in adipocytes, the possibility of calpains also having a role in the nuclear compartment of mammary epithelial cells during cell proliferation or progenitor differentiation is yet to be explored.

The role of calpains in proliferation of breast cancer cells has been more extensively studied. Depletion of *CAPN2* or *CAPNS1* by knockdown experiments in breast carcinoma cell lines reduced tumor growth in mouse orthotopic xenografts, (Ho et al., 2012; Grieve et al., 2016). Ablation of the regulatory subunit *CAPNS1* in the mammary epithelium delays spontaneous tumor onset in a model of mammary HER2^+^ tumorigenesis (MacLeod et al., 2018). Through the modulation of PP2A/Akt/FoxO3a pathway, *CAPN2* silencing induces the expression of cyclin-dependent p27Kip1 kinase inhibitor and reduces breast carcinoma cell proliferation (Ho et al., 2012). Furthermore, accumulation of nuclear calpain-2 has been associated to breast cancer cell proliferation. Nuclear calpain-2 has been observed in both, triple-negative and luminal breast cancer cell lines (Telechea-Fernández et al., 2018). *CAPN2* knockdown in triple-negative breast cancer cell lines causes a higher percentage of cells at G2/M, aberrant mitosis, fails in cytokinesis and consequently, an increased number of multinucleated cells (Rodríguez-Fernández et al., 2021). All these data suggest that while calpain-1, or both conventional calpains, participate in the differentiation program of adipocytes during mammary gland involution, calpain-2 preferentially accumulated in the nuclear compartment, seems to be the main isoform modulating cell proliferation in breast tumor cells.

However, the nuclear localization of calpain-2 has been described also in non-transformed epithelial cells. It has been reported that while in proliferating cells calpain-2 is mainly localized in the nucleus (König et al., 2003; Raynaud et al., 2004; Raynaud et al., 2008), in fully differentiated quiescent cells calpain-2 is restricted to the cytosol (Raynaud et al., 2004). High expression of *CAPN2* has been associated to its nuclear accumulation and active mitosis in ES cells as well as in 8-cell embryos (Raynaud et al., 2008). According to these data, calpain-2 might be expected to modulate cell proliferation and differentiation of epithelial cells in mammary gland during the pregnancy/lactation cycle. However, the effect of tissue and cell type in the physiological or pathological mammary gland needs to be considered. The physiological mammary stroma consists of adipocytes, fibroblasts, endothelial and inflammatory cells, as well as ECM, tightly regulated during each phase of the pregnancy/lactation cycle. The communication and interaction between the mammary epithelium and stroma drive the proper patterning and function of the normal mammary gland (Howard and Lu, 2014) and consequently, they will be determinant for the different functions of conventional calpains. Likewise, a malignant breast carcinoma includes more components than just epithelial tumor cells. Disruption of the above mentioned interactions or altered stroma composition in breast cancer (Landskroner-Eiger et al., 2010; Vizovisek et al., 2021) could alter the subcellular distribution and functions of conventional calpains in specific cell types. Although much progress has been made in understanding the function of conventional calpains on cell proliferation using breast cancer cells, these data highlight the need to consider the tissue composition and particular microenvironment when trying to elucidate the specific role and regulation of each calpain isoform.

## Challenges of calpains as therapeutic targets and concluding remarks

As we have extensively covered in this review, *in vitro* and *in vivo* experiments demonstrate that conventional calpains are involved in tumor progression and metastasis. Moreover, calpain inhibition has the potential to attenuate carcinogenesis and block metastasis of aggressive tumors and particularly, of breast cancer. Hence, targeting calpain-1 and calpain-2 was proposed as a novel therapeutic strategy for mammary tumors. However, there are not clinical trials involving inhibitors of conventional calpains for breast cancer treatment. One of the main reasons for failure in calpain-targeted chemotherapies is that calpains are a big family of proteases with more than 15 isoforms, and even when only limited to conventional calpains, the same isoform can have opposite effects in different tissues or cell types. Since it is difficult to dissect the isoform-specific role of both calpains for each particular cell context, the potential use of calpains as therapeutic targets is necessarily limited. In addition, when we consider that the role of each isoform might also depend on its subcellular location, targeting calpains seems an *a priori* unaffordable task.

Indeed, ablation of *CAPN2* in mice is embryonically lethal (Dutt et al., 2006) which further remarks the essential role of these proteases for the epithelial homeostasis. Consequently, the complete suppression of calpain activity can be harmful to the organism. However, conditional deletion of total or tissue-specific *CAPNS1*, which abrogates both conventional calpains, has been well tolerated and has been demonstrated to be a useful tool to unveil the essential role of calpains in maintaining tissue homeostasis (Grieve et al., 2016; MacLeod et al., 2018).

Recent advances in biomedicine and technology have further contributed to the design of new site-directed inhibitors of conventional calpains with promising effects on inhibition of the enzymatic activity. Nevertheless, many of these inhibitors, like the classical inhibitors ALLN or calpeptin, resulted not as specific as desired and the activity of other proteases such as cathepsins or caspases was also blocked (Shapovalov et al., 2022).

The main calpain inhibitors that have been disclosed over the last 10 years include different types of agents such as calpastatin-based peptidomimetics, thalassospiramide lipopeptides, disulfide analogs of α-mercaptoacrylic acids, allosteric modulators, azoloimidazolidenones and, macrocyclic/non-macrocyclic carboxamides (Donkor, 2020). All of them showing different characteristics, benefits, and disadvantages.

To name some of them, the peptidomimetics calpain inhibitors include agents based on the CAST structure. CAST is the only known endogenous inhibitor of conventional calpain (Kiss et al., 2008). Although other compounds can also inhibit other unconventional calpains like calpain-8 or -9, these agents inhibit mainly calpain-1 and -2 (Hata et al., 2016). Even though these peptides show higher specificity than others, they still show poor cell permeability and pharmacokinetic properties. As a result, a structure-guided design of isoform-specific inhibitors of calpains is yet to be accomplished.

The thalassospiramide are lipopeptides isolated from marine bacteria, found to inhibit human calpain-1 in the nanomolar range (Ross et al., 2013). These compounds have potential anti-inflammatory properties and exhibit low toxicity and good selectivity (Lu et al., 2015). Other compounds in this group include derivatives of the MG132, a tripeptide that inhibits both, 26S proteasome and calpain activity (Pehere et al., 2019). The detailed structure of the latter agents and the effectiveness of some of the inhibitors in preclinical animal models has been thoroughly discussed elsewhere (Donkor, 2020).

Having said that, there are some phase II/III and even preclinical studies with calpains inhibitors, but they have been tested in the context of other diseases, such as Alzheimer’s disease, multiple sclerosis, spinal muscular atrophy, traumatic brain injury, acute myocardial infarction, ophthalmic diseases, or muscular dystrophy (Ono et al., 2016). As commented in this review, the calpain system has been predicted to be an important target for cancer treatment (Miyazaki et al., 2015). In that sense, calpain-1 activity was shown to be important in the treatment of other types of cancer such as myelodysplastic syndrome (Fang et al., 2016) colorectal cancer (Vaish and Sanyal, 2012) or melanoma (Del Bello et al., 2007). However, although the latter reports show the initial benefits of inhibiting conventional calpains, other studies in melanoma have suggested that calpain activity is required for the success of cisplatin-induced apoptosis of cancer cells (Moretti et al., 2014).

In summary, as we have highlighted throughout this review, conventional calpains can have different or even opposite functions in different cell types or biological contexts. It is noteworthy to mention that a scarce number of reports in the literature show *in vivo* models for the study of conventional calpains in physiological or pathological mammary gland. Even more, while a limited number of publications show studies in breast cancer cell lines, most of reports on the role of conventional calpains do not use mammary cells as experimental models. Consequently, the mechanisms of regulation of calpain activity in mammary tissue are still unknown. Indeed, it is not known how conventional calpains are regulated to specifically recognize a substrate among all the proteins known to be their targets in mammary tissue. Although the effect of calpains have been studied in breast cancer or mammary gland involution, the mechanisms of calpain activation have not been studied and only hypothetical and not demonstrated connections between regulatory factors and calpains can be found in the literature.

On the other hand, the subcellular compartmentalization of calpains, which limits their access to substrates, seems to be a key event for their functions. Thus, understanding the mechanisms underlying subcellular distribution of calpains will be crucial to decipher or inhibit their functions. A major challenge in targeting conventional calpains as a therapeutic approach for breast cancer would be to specifically abolish a calpain isoform within a cell compartment and cell type in mammary tissue. Hence, important questions to be answered are: How are conventional calpains differentially distributed into cell compartments in breast cancer cells? Might the subcellular localization of calpain isoforms, instead of the expression levels of calpains or calpastatin, have a prognosis value in breast cancer? In the meanwhile, post-lactation mammary gland involution seems to be the most useful model to answer those questions. Pregnancy associated breast cancer has been explained as the progression of a pre-existing disease promoted by the microenvironment of post-lactating mammary gland (Macdonald, 2020). If involuting mammary gland mimics the microenvironment of a developing tumor, unraveling the multifaceted and isoform-specific roles of calpains in the context of mammary gland involution will lead us to gain insights into breast cancer development and the design of new calpain-targeting therapies.
